# Investigating the impact of electrical stimulation temporal distribution on cortical network responses

**DOI:** 10.1186/s12868-017-0366-z

**Published:** 2017-06-12

**Authors:** Francesca Scarsi, Jacopo Tessadori, Michela Chiappalone, Valentina Pasquale

**Affiliations:** Department of Neuroscience and Brain Technologies (NBT), Istituto Italiano di Tecnologia (IIT), Via Morego 30, 16163 Genoa, Italy

**Keywords:** Cortical network, Electrical stimulation, Micro-electrode array, Scale-free, Noise, Response reliability

## Abstract

**Background:**

The brain is continuously targeted by a wealth of stimuli with complex spatio-temporal patterns and has presumably evolved in order to cope with those inputs in an optimal way. Previous studies investigating the response capabilities of either single neurons or intact sensory systems to external stimulation demonstrated that stimuli temporal distribution is an important, if often overlooked, parameter.

**Results:**

In this study we investigated how cortical networks plated over micro-electrode arrays respond to different stimulation sequences in which inter-pulse intervals followed a 1/*f* ^*β*^ distribution, for different values of *β* ranging from 0 to ∞. Cross-correlation analysis revealed that network activity preferentially synchronizes with external input sequences featuring *β* closer to 1 and, in any case, never for regular (i.e. fixed-frequency) stimulation sequences. We then tested the interplay between different average stimulation frequencies (based on the intrinsic firing/bursting frequency of the network) for two selected values of *β*, i.e. 1 (scale free) and ∞ (regular). In general, we observed no preference for stimulation frequencies matching the endogenous rhythms of the network. Moreover, we found that in case of regular stimulation the capability of the network to follow the stimulation sequence was negatively correlated to the absolute stimulation frequency, whereas using scale-free stimulation cross-correlation between input and output sequences was independent from average input frequency.

**Conclusions:**

Our results point out that the preference for a scale-free distribution of the stimuli is observed also at network level and should be taken into account in designing more efficient protocols for neuromodulation purposes.

**Electronic supplementary material:**

The online version of this article (doi:10.1186/s12868-017-0366-z) contains supplementary material, which is available to authorized users.

## Background

Neurons are intrinsically noisy systems, as stochastic processes regulate neuronal function at many different spatial and temporal scales, from genetic and metabolic noise all the way up to firing activity on the scale of the whole brain [1–3]. This evidence suggests that noisy stimulation might be far more effective at driving neuronal networks because it mimics natural sensory input. In fact, variability of neural responses to stimulation is reduced if sensory input is natural or natural-like [4–9]. Spike trains of sensory neurons tend to become more reliable if inputs present a scale-free structure, akin to that observed in most natural signals [10–12]. Similarly, isolated neurons respond with unpredictable patterns when presented with constant inputs but observed responses become almost perfectly reproducible if the neuron is stimulated with a natural-like signal [13, 14]. Early works focused on short timescales (i.e. a few seconds); more recently, it has been demonstrated that even at longer timescales single neuron responses synchronize with input sequences, as long as those sequences present a scale-free temporal structure [15]. Irregular stimulation protocols have been recently also proposed in the context of deep brain stimulation [16], aimed at improving current clinical outcomes in the treatment of pathologies (e.g. stroke) that benefit from the application of targeted electrical stimulation.

In this paper we aimed at filling the gap in scale between complete systems (i.e. whole brain) and isolated neurons by investigating the response patterns of neuronal networks when presented with input sequences exhibiting different temporal structures. To this end, we used primary cortical cultures grown over micro-electrode arrays (MEAs). MEA devices, introduced at the end of the 70s [17] allow to record and stimulate a neuronal network at many different locations at once and to observe it over long timescales [18]. Dissociated cortical cultures retain several interesting functions of the original brain tissue, and, during in vitro development, they start showing spontaneous activity at about 7 days in vitro, DIVs [19–21]. Then, network activity gradually changes, first as single spikes gather into bursts, generally towards the end of the second week in vitro, and later featuring a highly complex pattern of synchronized, aperiodic network bursts [22–24], which represents the mature state of a cortical network.

By taking advantage of MEA technology, we designed two experimental protocols, based on electrical stimulation of cortical cultures, aimed at answering two main scientific questions. Specifically, we first asked whether cortical networks’ activity is more easily entrained by irregular sequences of stimulus pulses than by regular sequences, as it happens for isolated neurons [15]. To this end, we designed stimulation sequences in which inter-pulse intervals followed a 1/*f* ^*β*^ distribution, for different values of *β*, ranging from 0 (i.e. corresponding to white noise with no correlation in time) to ∞ (i.e. fixed-frequency). As proposed in the paper by Gal and Marom [15], we measured the correlation between instantaneous stimulation and firing frequencies to quantify the network capability to follow different temporal patterns of pulses. Furthermore, we investigated the interplay between stimulation frequency and stimulation regularity to understand whether regular and irregular sequences of stimuli would present the same response profiles, at different average stimulation rates.

## Methods

### Neural preparation

As experimental model for our research, we used primary cortical cultures from embryonic rats. All experimental procedures and animal care have been approved by the IIT Animal Welfare Body and by the Italian Ministry of Health (authorization 110/2014-PR), in accordance with the National Legislation (D.Lgs. 26/2014) and the European legislation (European Directive 2010/63/EU).

**Fig. 1:**
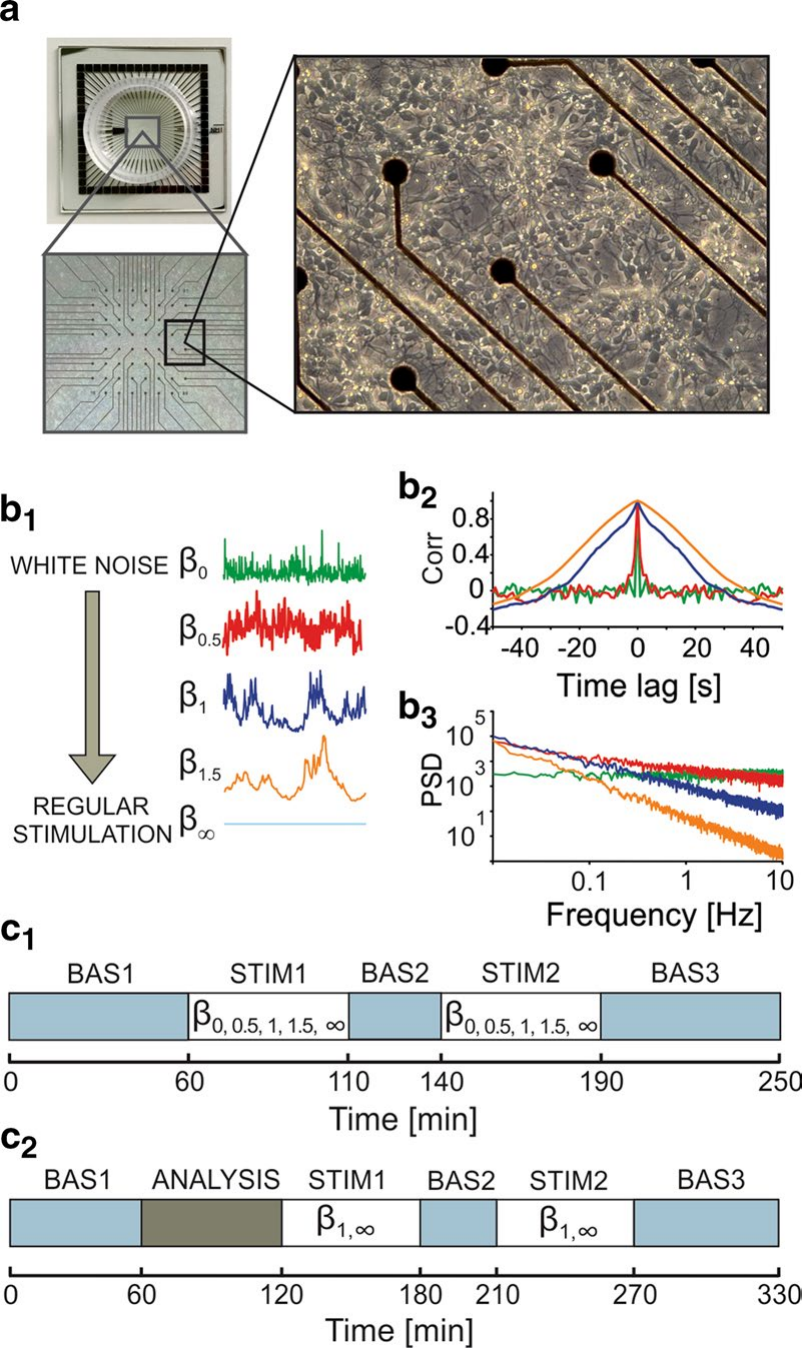
Experimental model and stimulation protocols. **a** Cortical neuronal network grown over a micro electrode array (MEA). *Top left* A typical 60-channel MEA produced by multichannel systems (MCS, Reutlingen, Germany). The glass ring is necessary to contain cell culture medium. *Bottom left* Dissociated cortical neurons over substrate-embedded planar microelectrodes of a MEA. The electrodes, placed in a square 8 × 8 layout (the four corners are missing), are 30 µm wide, with an electrode spacing of 200 µm. *Right* Zoom of the cultured cortical network showing cells coupled to microelectrodes and randomly developing their neurites and synaptic connections (age of the culture: 21 days in vitro, DIV). **b** Stimulation regimes. *b1* We used trains of electrical pulses whose time intervals followed a 1/*f* ^*β*^ distribution, with β assuming values from 0 (i.e. white noise stimulation) to ∞ (perfectly regular stimulation). Depicted here are the profiles of the instantaneous stimulus rate (ISR, see text—rescaled for comparison) for each value of β: β = 0 (*green trace*), β = 0.5 (*red trace*), β = 1 (*blue trace*), β = 1.5 (*orange trace*) and β = ∞ (*light blue trace*). Autocorrelation functions (*b2*) and PSD (*b3*) of irregular stimulation sequences (same *color code* as in *b1*). **c** Adopted experimental protocols. *c1* 1st experimental protocol (protocol 1 throughout the manuscript): three phases of spontaneous activity [BAS1—1 h, BAS2—30 min and BAS3—1 h] are interleaved by two stimulation sessions [STIM1 and STIM2] lasting 50 min (i.e. 10 min × 5 different β values [0, 0.5, 1, 1.5, ∞], delivered in random order). *c2* 2nd experimental protocol (protocol 2 throughout manuscript): three phases of spontaneous activity [BAS1—1 h, BAS2—30 min and BAS3—1 h] are interleaved by one session of off-line analysis [ANALYSIS] and two stimulation sessions [STIM1 and STIM2] lasting 60 min (i.e. 10 min × 2 different β values [1 and ∞] × 3 stimulation frequencies [f_MBR/2_, f_MBR_, f_MFR_]. *For details* see paragraph 2.3 “Generation of stimuli and experimental protocols”)

The procedures for preparing and maintaining neuronal cultures were described in details in previous studies from our group [25]. Briefly, embryos were recovered from CO_2_-anaesthetized pregnant rats at embryonic day 18 (Sprague–Dawley derived by Charles River in 1955, IGS). The cortices were then exposed to chemical and mechanical dissociation. Afterwards, cells were plated onto 60-channel MEA devices (Fig. 1a, top left panel) previously coated with poly-d-lysine and laminin, at the final concentration of 1500 cell/µl (Fig. 1a, bottom left panel). They were maintained on MEA devices containing 1 ml of nutrient medium (i.e. serum free Neurobasal medium supplemented with 2% of B27, 1% of Glutamax and 0.04% of Gentamicin), in a humidified incubator with a controlled atmosphere of 5% CO_2_–95% air at 37 °C. The cultures were kept in the incubator until they reached a mature stage of the development, around 3–4 weeks in vitro (Fig. 1a, right panel), as reported in previous studies [22]. Half of the medium was changed weekly.

In our cortical cultures we have percentages of cells similar to what found in the intact cortex (i.e. 70–80% excitatory and 20–30% inhibitory neurons [26]), as reported by previous studies [27–29]. It is worth underlining that an important (and fundamental) element of our cultures is the presence of glial cells. In our cultures, neurons and glia grow together and these environmental conditions allow cortical neurons to show excellent growth and robust synaptic connectivity [22].

### Experimental set-up

As stated in the previous paragraph, we used MEAs as recording devices. In the MEA, planar microelectrodes are arranged in an 8 × 8 layout excluding corners and one large ground electrode, for a total of 59 recording electrodes (30 μm diameter, 200 µm inter-electrode distance).

The experimental set-up is based on the MEA 60 system, consisting of the MEA itself containing the neural preparation, a mounting support with integrated 60-channel preamplifiers and filters, a stimulus generator (STG 2004) and a desktop computer with the software tools MC_Rack and MC_Stimulus, which were used for on-line signal visualization, raw data recording and stimulus sequence delivery. All the mentioned devices and software are commercially available and produced by MultiChannel Systems, Reutlingen, Germany. Each recorded channel was sampled at a frequency of 10 kHz.

To reduce thermal stress of the neurons during each experiment, MEAs were kept at 37 °C by means of a controlled thermostat (MultiChannel Systems) and covered by a custom–made poly(dimethylsiloxane) cap to avoid evaporation and to prevent changes in medium’s osmolarity. Additionally, we have settled a custom chamber to maintain a controlled atmosphere (i.e. gas flow of 5% CO_2_ and 95% O_2_ + N_2_) during the entire recording time, as reported in previous papers [30].

### Generation of stimuli and experimental protocols

In order to test the behavior of cultures when provided with inputs of varying regularity, stimulation trains with an increasing degree of regularity had to be designed. To this end, we took advantage of the concept proposed in literature in previous papers [15, 31, 32]. Our stimulation sequences consisted of trains of electrical pulses whose time intervals followed a 1/*f* ^*β*^ distribution. Intuitively, an increase in the parameter *β* resulted in an increase of the regularity of the corresponding sequence: for *β* = 0, subsequent intervals were uncorrelated; for 0 < *β* < ∞, neighboring time intervals presented an increasing amount of correlation and *β* = ∞ resulted in a perfectly regular sequence (i.e. time intervals have all the same length). More in detail, the inter-pulse interval series presented in Fig. 1b result from the five different values of *β* considered in this work (0, 0.5, 1, 1.5 and ∞, represented with green, red, blue, orange and cyan curves). The autocorrelation functions of the stimulation trains and their power spectral densities (PSD) are reported in Fig. 1, panels b2 and b3 respectively. The basic stimulus used has remained constant throughout all experiments (biphasic voltage pulse, 300 µs in half-length phase and 750 mV of half-amplitude). We made this choice following the results presented by Wagenaar [33]: according to his study, those parameters were the most effective in evoking responses in cultured neurons on MEAs.

The stimulation trains described above are entirely defined by the sequence of inter-pulse intervals: in order to generate the stimulation patterns, interval sequences with the proper features had to be produced. In particular, a sequence of N stimuli is defined by the length of the (N − 1) intervals between them. Therefore (N − 1) uncorrelated samples are extracted from a Gaussian distribution. This sequence is then filtered in the frequency domain with an appropriate filter (i.e. a signal whose PSD is a line with slope −*β* in a log–log plot). The resulting sequence then undergoes a linear transformation to ensure that standard deviations and mean values match with the desired specifications. All the stimulus sequences have been generated by a custom script developed in MATLAB^®^ (The Mathworks, Natick, MA, USA).

We performed two experimental campaigns, according to the main scientific questions we wanted to address (cf. “Background”), for a total of 17 experiments between 22 and 35 DIVs. The first experimental protocol, named ‘protocol 1’, graphically depicted in Fig. 1c1, had a total duration of 250 min, which were divided in the following recording phases:

- Phase 1: 1 h of spontaneous activity recording [BAS1];
- Phase 2: 50 min of electrical stimulation from a selected channel, chosen from those that were active and that evoked responses in the culture. This step is further divided into 5 sub-phases of 10 min each, one for each value of the parameter β, delivered in random order [STIM1];
- Phase 3: 30 min of spontaneous activity recording [BAS2];
- Phase 4: 50 min of electrical stimulation, repeating phase 2 [STIM2];
- Phase 5: 1 h of spontaneous activity recording [BAS3];

All stimulation sequences were designed according to the procedure described above in order to have an average inter-stimulus interval of 2 s and, where applicable (i.e. sequences with *β* < ∞), a standard deviation of 0.5 s. We performed a total of 9 experiments by using this protocol. In a set of pilot control experiments, we delivered the same stimulation protocol from two different channels: preliminary results indicated no significant differences between the two, so we decided to test a single channel per culture in the final set of experiments.

To respond to our second scientific question, we designed a second experimental protocol, named ‘protocol 2’ and graphically depicted in Fig. 1c2, which had a total duration of 330 min, which were divided in the following recording phases:

- Phase 1: 1 h of spontaneous activity recording [BAS1];
- Phase 2: About 1 h reserved for analysis. During this time, spontaneous activity of the culture is analyzed (cf. “Data analysis”—“‘On-the-fly’ analysis for protocol 2”), [ANALYSIS];
- Phase 3: 1 h of stimulation from an active channel. Six stimulation sequences are delivered during this time. Those sequences alternate in regularity, between *β* values of ∞ and 1, and present an increasing stimulation rate: the first pair of sequences has an average inter-stimulus interval equal to half the average inter-burst period observed in the spontaneous recording (i.e. mean stimulation rate—MSR equal to f_MBR/2_, where MBR stands for mean bursting rate), while the second and third pairs of stimulation sequences have an inter-stimulus interval matching, respectively, the computed average bursting rate (MSR = f_MBR_) and the mean firing rate—MFR (MSR = f_MFR_), [STIM1];
- Phase 4: 30 min of spontaneous activity recording [BAS2];
- Phase 5: 60 min of electrical stimulation, repeating phase 3 [STIM2];
- Phase 6: 60 min of spontaneous activity recording [BAS3];

While average inter-stimulus intervals varied as described above, standard deviations were kept constant at 0.5 s. We performed a total of 8 experiments using this protocol.

### Data analysis

Data analysis was performed off-line through MATLAB, using an ad-hoc developed software package named SpyCode [34]. It provides a working environment able to perform efficient data management and processing since it includes a rich group of common signal analysis tools. Briefly, raw data from the acquisition system were imported in MATLAB and spike detection was performed using the Precise Timing Spike Detection algorithm [35]. Once spikes have been identified, we computed, for each recording, the mean firing rate (MFR, expressed as number of spikes/s), a first-order statistical indicator that quantifies the average firing frequency in a specific time window. We used this parameter to evaluate the level of electrophysiological activity of the network during the basal ‘spontaneous’ phases (i.e. BAS1, BAS2, BAS3 in Fig. 1c1, c2). MFR values of each experiment were normalized with respect to the population average computed during the first spontaneous phase (i.e. BAS1) [21, 36]. Then, in order to detect and evaluate the average effect of a stimulation pattern on the spike activity, we computed the post-stimulus-time histogram (PSTH) [37, 38]. Intuitively, the PSTH shows the probability of firing (or the instantaneous firing rate) as a function of time in a short window after stimulus onset.

Cultured neurons, in addition to individual spikes, also exhibit more complex patterns of activity called bursts, which are dense sequences of closely packed spikes [39, 40]. The bursting behavior changes along the in vitro development reaching stability between the third and fourth week [22]. Bursts come in different forms, so the bursting rate is not sufficient to describe the “burstiness” level of a culture [41]. To this end, during the spontaneous activity phases (i.e. BAS1, BAS2, BAS3), we quantified the number of spikes within bursts. The ‘spike in burst’ parameter was calculated as the ratio between the number of spikes within bursts and the total number of spikes for each recording session. Burst detection was performed by using an algorithm based on ISI criteria developed in our lab a few years ago [42], which has been recently recognized as one of the most reliable available in the literature [43].

To quantify the global variability of responses during different stimulation regimes (i.e. β values), we computed the local variation with refractoriness (LvR) parameter, which has been successfully applied to classify firing patterns based on (ir)regularity and it is not confounded by the firing rate [44].

#### ‘On-the-fly’ analysis for protocol 2

For the second protocol, we performed an ‘on-the-fly’ analysis aimed at evaluating the basic statistics in terms of firing and bursting activity of the measured culture. The obtained results were then used to define the MSR to be used in the course of the experiment (cf. “Generation of stimuli and experimental protocols”). Specifically, we performed two analyses. First we computed the MFR of the first hour of spontaneous activity of the culture. The obtained value was used as MSR for the sequence of stimulation referred to as f_MFR_. On the same recording, we performed network burst detection, following the algorithm developed by van Pelt et al. [45, 46]. Intuitively, a network burst consists of synchronized activity occurring simultaneously on most of the active channels of the MEA. In particular, van Pelt’s method is based on the assumption that, during a network burst, both firing rate in individual channels and number of active sites increase. More in detail, this algorithm consists in splitting spike trains of all recorded channels in 25 ms-long bins. Each time bin is then attributed a ‘bursting value’ equal to the product of the total number of spikes detected within each bin and the number of electrodes recording at least 1 spike within the same time interval. This value is then compared to a user-defined threshold and a network burst is identified each time the product defined above exceeds this threshold. In our case, we use thresholds around or above 100 depending on network activity [22]. Once the network bursts were detected, we calculated the rate of occurrence of those events and designed two sequences of stimuli with MSR equal to the average network burst rate (i.e. f_MBR_) and half of that (i.e. f_MBR/2_), respectively.

#### Correlation analysis

In order to evaluate the correlation between the firing profile of a network and the stimulation profile delivered during each experimental session, spike trains were analyzed to compute the ‘network-wide firing rate’. This signal is obtained by computing the sum of all spike trains to obtain a cumulative spike train, and then by binning it at 1-ms time resolution (cf. Additional file 1: Figure S1A).

The resulting signal was low-pass filtered to obtain the instantaneous firing rate (IFR). Specifically, the filters used in this work are Gaussian windows of width T and *α* equal to 2.5 (where *α* is defined as the reciprocal of the standard deviation). A constant value is then subtracted from all coefficients to obtain a mean value equal to zero. Four different window lengths T have been tested to investigate behaviors at different time scales: 2, 4, 8 and 16 s. Stimulation trains are filtered in the same manner to obtain the instantaneous stimulation rate (ISR). We then used cross-correlation analysis to evaluate the effect of local stimulation on the evoked spiking activity of the network: we computed the I/O correlation (i.e. the cross-correlation between ISR and IFR), for different values of *β* and for different filter lengths. A slight variant of Pearson’s coefficient (PC) has been used to evaluate I/O correlation: since response delay to stimulation was unknown, we took as PC the maximum value of normalized cross-correlation in a 2-s window across its 0-lag point, instead of the 0-lag value itself as per standard definition. For simplicity, this measure has been addressed as Pearson’s coefficient in the rest of the paper, even though it is a slight misnomer. Most of the figures in this paper report IFRs and ISRs in arbitrary units: as they are only used to compute Pearson’s coefficient, the actual scale is irrelevant.

Each experiment performed with protocol 1 resulted in 2 series of 20 values of PC (combination of 5 values of β and 4 values of T, while each series was obtained for a repetition of the stimulation sequences—cf. “Generation of stimuli and experimental protocol”). For protocol 2, each experiment provided 2 series of 24 values of PC (3 regular stimulation sequences, 3 irregular stimulation sequences, each recording analyzed with 4 filter windows. 1 series obtained for each repetition).

#### Statistics

All data presented are expressed as mean ± standard error of the mean, if not differently specified. All box plots represent mean (small square), median (line), 25th–75th percentiles (box), and 5th–95th percentiles (whiskers). Statistical tests were employed to evaluate the significant difference among different experimental conditions. The normal distribution of experimental data was assessed using the Shapiro–Wilk normality test (p level 0.05). According to the distribution of the data, we performed either parametric (e.g. t test, paired t test, ANOVA) or non-parametric (e.g. Kruskal–Wallis ANOVA on ranks, Mann–Whitney U test) tests and p values <0.05 were considered significant. The post hoc Bonferroni (for the ANOVA) or Tukey’s (for the Kruskal–Wallis) test was used to assess differences among multiple conditions. Statistical analysis was carried out by using either OriginPro (OriginLab Corporation, Northampton, MA, USA) or SigmaPlot (Systat Software, Inc. San Jose, CA, USA).

## Results

### Time-averaged responses are not affected by temporal distribution of stimuli

In this paper, we used dissociated cortical cultures plated over MEAs in order to understand whether and how temporal regularity affects the network capability to follow an external stimulation. To reach our objective, we designed two experimental protocols (i.e. protocol 1 and protocol 2, cf. “Methods”, “Generation of stimuli and experimental protocols”) aimed at answering two main scientific questions: (1) Can we find a preference for stimulus irregularity at the network level? (2) If such a preference exists, is it affected by the average stimulation frequency?

We first evaluated the level of firing and bursting of our networks by computing MFR and the spike in burst ratio during the three spontaneous activity recording phases of each protocol (i.e. BAS1, BAS2, and BAS3). No statistical difference was observed (cf. Additional file 1: Figure S1, panel B for protocol 1 and panel C for protocol 2), indicating that the network activity of our cultures was stable all along the experimental time. Moreover, no short/medium-term effect on the level of firing and bursting was induced by our stimulation sequences.

**Fig. 2:**
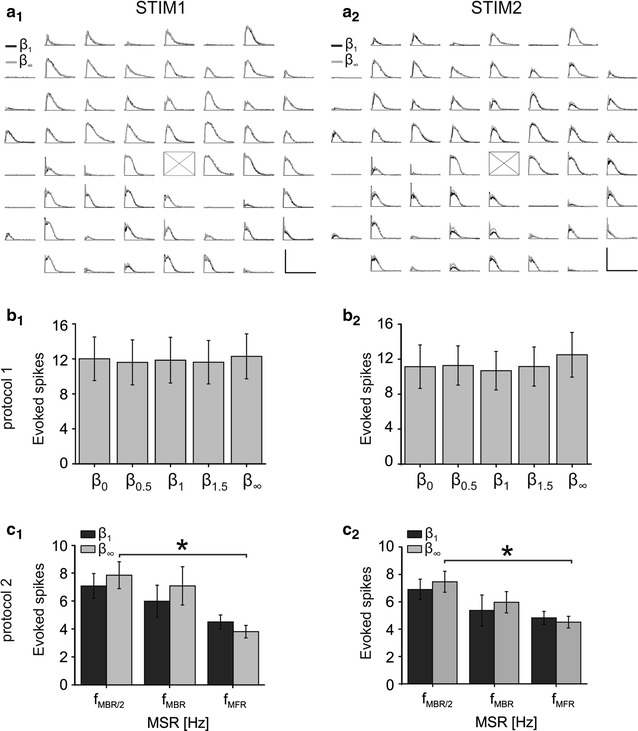
Analysis of evoked activity for the two designed protocols. **a** PSTH map of sample experiment during irregular (β = 1, *black trace*) and regular (β = ∞, *gray trace*) stimulation, delivered from channel 55, marked with an ‘X’ on the map. *a1* and *a2* show respectively the PSTH profiles observed during the first (STIM1) and second (STIM2) repetition of the same stimulation protocol. *Scale bars* for *a1* and *a2*. *X axis* Time [0–400 ms]; *Y axis* Spike Count [0–2]. **b** Computation of the average number of evoked spikes during the two stimulation sessions of protocol 1. *b1* and *b2* show the mean number of evoked spikes computed for all the performed experiments, respectively, during first and second repetition, i.e. STIM1 and STIM2, with varying values of β used during the stimulation session (i.e. β = 0, β = 0.5, β = 1, β = 1.5, β = ∞). No statistical difference is observed among the five conditions (p > 0.05, One-way ANOVA, post hoc Bonferroni’s method). **c** Computation of the average number of evoked spikes during the two stimulation sessions of protocol 2. *c1* and *c2* show, respectively, the mean number of evoked spikes computed for all the performed experiments during first and second repetitions of protocol 2, with varying values of β (i.e. β = 1, black bars; β = ∞, light gray bars) and mean stimulation rates (MSR): f_MBR/2_ (0.07 ± 0.04, mean ± SD), f_MBR_ (0.14 ± 0.08 Hz, mean ± SD), f_MFR_ (0.54 ± 0.34 Hz, mean ± SD), respectively equal to half MBR, MBR and MFR obtained with an on-the-fly analysis of the first hour of the network’s spontaneous activity. A significant statistical difference was observed for β = ∞ between the number of evoked spikes obtained during regular stimulation at f_MBR/2_ and f_MFR_ (*p < 0.05, One-way ANOVA, post hoc Bonferroni’s method) for both repetitions. Data used to compute the statistical distributions reported in (**b**) and (**c**) are included in Additional file 2

Figure 2 presents data relative to PSTH analysis (cf. Additional file 2). In particular, from the upper panels of Fig. 2 (panels a1 for STIM1 and a2 for STIM2), it is possible to note how averaged network responses following irregular stimulation (in this case, for *β* = 1, black trace) show a profile that is qualitatively very similar to what can be observed after fixed-frequency stimulation (*β* = ∞, light gray trace). Furthermore, the PSTH profiles relative to the first (Fig. 2a1) and second (Fig. 2a2) stimulation phases, are almost identical, suggesting that responses do not change on average upon the application of short periods of low-frequency stimulation. This is reinforced by quantitative population analysis shown in the following panels of Fig. 2. In Fig. 2b1 (STIM1) and b2 (STIM2) no statistically significant difference is observed among the average number of evoked spikes obtained for different *β* values (p > 0.05, one-way ANOVA, post hoc Bonferroni correction). Additionally, no difference was found between STIM1 and STIM2 for protocol 1 for all values of *β* (p > 0.05, paired t test). A similar observation can be made for Fig. 2c, relative to protocol 2. The number of evoked spikes does not change as a function of stimulation regularity, both during STIM1 (panel c1) and STIM2 (panel c2), i.e. no significant differences between β = 1, dark bars, and β = ∞, light gray bars, for STIM1 and STIM2; no significant differences between STIM1 and STIM2 for each β value, p > 0.05 paired t-test. We also computed the LvR of each responsive channel in each stimulation regime. We did not find any significant difference among the values of ß, meaning that the average variability of response firing patterns is constant in the different conditions. We performed this analysis for both STIM 1 and STIM 2 and obtained the same results (see Additional file 1: Figure S2).

On the other hand, a change in the average MSR causes a change in the observed response amplitude, which results to be significant only in case of regular stimulation (i.e. β = ∞): longer inter-stimulus intervals lead to more intense responses, on average. These results provide evidence that the average response amplitude to stimulation is largely unaffected by stimulation regularity. In the following, we will investigate whether the distribution of responses in time, conversely, is affected by stimulation regularity.

### Network activity better follows scale-free than regularly distributed stimuli

**Fig. 3:**
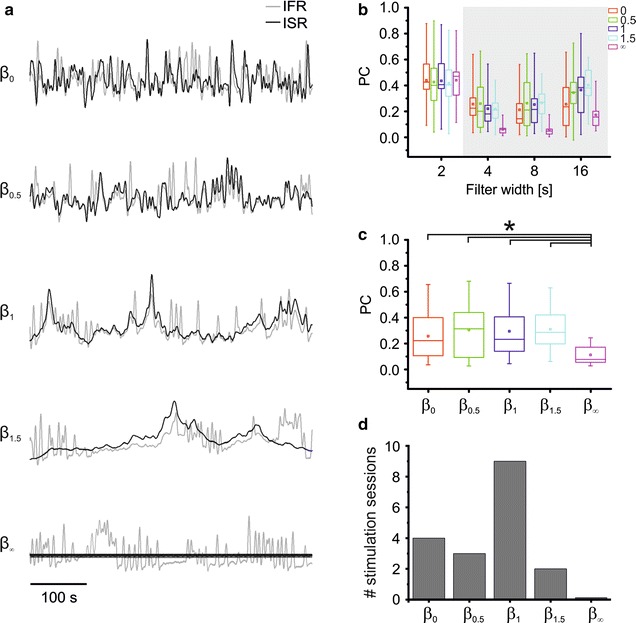
Input and output correlations under the five stimulation regimes during the first stimulation session of protocol 1. **a** Instantaneous firing rate (IFR, *gray trace*) and instantaneous stimulation rate (ISR, *black trace*) of a representative experiment under the five stimulation regimes during phase STIM1 of protocol 1. 500 s of activity are reported. Traces are normalized with mean = 0 and std = 1 for ease of comparison. *Y-axis* is in arbitrary units. *Scale bar* 100 s. **b** Box plots of Pearson’s coefficients (PCs) between ISR and IFR for all tested β values as a function of the filter length T (nine experiments, STIM1). **c** Box plots of PCs computed for each *β* value, regardless of the filter length used (i.e. each box plot represents 27 values − 9 experiments × 3 filtering window lengths, T = (4, 8, 16) s). Asterisks denote populations whose median values are statistically different (*p < 0.05, Kruskal–Wallis test, post hoc Tukey’s method). **d** ‘Preferred’ *β* values for Protocol 1. The *bar graph* reports the number of stimulation series (nine cultures, two sessions per culture) resulting in the top PC score for each *β* value tested. Data used to compute the statistical distributions reported in (**b**), (**c**) and (**d**) are included in Additional file 2

Figure 3a shows an example of an experiment conducted with protocol 1. Panels in Fig. 3a display the IFR and ISR traces (respectively, in gray and black) for the central 500 s of recordings. Note that 30 s of recordings at the beginning and at the end of the stimulation sequence have been removed from representation and analysis to exclude border effects. In particular, the presented graphs are traces obtained after filtering the cumulative spike trains with a 8 s-long Gaussian window, as described in “Methods” (cf. “Data analysis—Correlation analysis”). Comparable results have been obtained for the second stimulation repetition (data not shown). As it can be easily observed from the represented traces, IFRs generally follow stimulation traces, with two major differences: regular stimulation (bottom panel) does not seem to be able to evoke similarly regular responses from the network and it can be clearly seen how the evoked activity follows an erratic pattern; the other difference is the presence, in the IFR traces, of sharp, tall peaks. These sudden increases in the network-wide firing rate correspond to intense synchronized bursting events, typically occurring in mature cortical cultures as reported by many studies in the past [20, 22, 45, 47, 48]. Differently from the methodological approach followed by Gal and Marom [15], we did not use any pharmacological cocktail to inhibit synaptic transmission in our cultures, thus spontaneous activity was still present when stimulating.

Population results for the nine experiments performed with this protocol are presented in Fig. 3b, c (cf. Additional file 2). Figure 3b reports the statistical distributions of PCs for each value of tested *β* as a function of the length of the filter used. The shortest filter window used, 2 s, results in similar PC values regardless of *β* value. The 2 s-filter is the only one for which this occurs: this result is likely due to the fact that only a single stimulus, on average, lies within the filter width (the average inter stimulus interval is, in fact, exactly 2 s). This result intuitively matches the fact that average response amplitudes, as quantified by the total number of evoked spikes in a short window following each pulse (cf. Fig. 2), are not affected by stimulation regularity. If the filter length increases above the average inter-stimulus interval, the observed PC values start to change as a function of stimulation regularity and repetition: perfectly regular stimulation fails to evoke matching responses, resulting in the lowest scores of PC in almost all tested cultures. Too much irregularity (i.e. *β* = 0) causes a similar though weaker effect, with PC scores ranking second-lowest for filter lengths of 8 s and 16 s. The central tested values of *β* (0.5, 1 and 1.5) result in similar PC values.

Figure 3c presents the same data as the panels above regardless of filter length used (excluding T = 2 s): each box plot displays the distribution of PC scores obtained in the 9 performed experiments at T = (4, 8, 16) s (i.e. each box plot represents a grand total of 27 points). We excluded T = 2 s for the considerations mentioned above (i.e. this filter length does not provide meaningful results in this context). When considering all timescales altogether, all *β* values result in higher correlations between input and output signals than regular sequences, and the difference is statistically significant. *β* = 0 leads to a middle-ground situation, with PC values not significantly different from any of the other *β* values (0.5, 1 and 1.5), but significantly different from the regular one. However, *β* values of 0.5, 1 and 1.5 tend to give higher PC values for longer timescales (T = (8, 16) s, see Fig. 3b) than *β* = 0.

The number of stimulation series (i.e. 2 for each of the 9 tested cultures, after Gaussian 8-s pre-filtering) resulting in the highest PC scores, for each value of *β*, have been computed. We reported in Fig. 3d results coming from both STIM1 and STIM2, since we found that there is no significant difference between the two sessions regarding the performed analyses. As it is possible to observe, the most frequent value to result in the highest I/O correlation is 1, but different cultures show different behaviors, with values of *β* ranging from 0 to 1.5 resulting in highest PC scores.

### Stimulation rate has no influence if the stimulus distribution is scale-free

We asked whether the average stimulation frequency (which in protocol 1 had been fixed to 0.5 Hz independently of the spontaneous network dynamics) had an influence on the temporal distribution of responses, and in particular we wanted to test whether average stimulation frequencies matching the endogenous rhythms of the network resulted in higher stimulus–response correlations. Given that network activity is better entrained by scale-free distributed stimuli (i.e. $\beta \cong 1$) than by fully regular stimuli (i.e. *β* = ∞) (cf. Fig. 3), we decided to focus our attention on *β* = 1 and *β* = ∞, by varying the average stimulation frequency in a range comprised between f_MBR/2_ (i.e. half of the spontaneous bursting frequency) and f_MFR_ (i.e. mean spontaneous firing rate) (cf. “Methods: “Data analysis”—“‘On-the-fly’ analysis for protocol 2”). This gave us also the possibility to check whether the capability of the network to follow increasing stimulation frequencies is affected by the temporal distribution of stimuli themselves. Hence we designed a second protocol (i.e. protocol 2), consisting in delivering stimulation sequences with either *β* equal to 1 or ∞ and different average inter-pulse intervals.

Analysis of collected data indicates that there appears to be no relation between MFR/MSR ratio (or MBR/MSR) and I/O correlation (data not shown): stimulation frequencies matching either spontaneous firing or spontaneous bursting rates seem to have no relevance on the evoked response pattern. On the other hand, absolute levels of MSRs proved to be more interesting: all stimulation phases have been divided in two groups based on their MSR; the threshold between the two groups was arbitrarily set at 0.2 Hz. In particular, this value was chosen as it is generally used as the maximum stimulation frequency for which independent responses can still be expected [41].

**Fig. 4:**
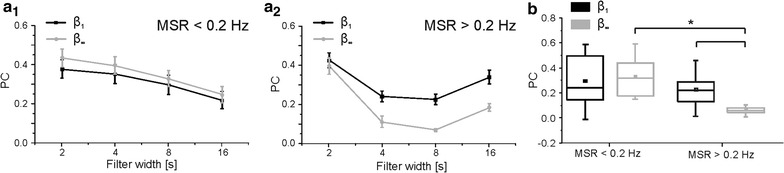
Input and output correlations for population during first stimulation session of protocol 2. **a** Panels show the mean (*dots*) and standard deviation (*error bars*) of the PCs computed with different filter lengths. *First panel* (*a1*) depicts results obtained on stimulation sequences with average repetitions slower than 0.2 Hz, *second panel* (*a2*) for those with stimulation frequency greater than 0.2 Hz. **b** Population statistics of all the PCs regardless of the filter length used for the stimulation sequences delivered. *Asterisks* denote populations whose mean values are statistically different (*p < 0.05, Mann–Whitney test). Data used to compute the statistical distributions reported in panels (**a**) and (**b**) are included in Additional file 2

Figure 4 present quantitative population results for all the experiments performed with protocol 2 (cf. Additional file 2). The two panels of Fig. 4a present the PCs obtained for the two groups (MSR < 0.2 Hz and MSR > 0.2 Hz) for increasing values of the filter width and different values of *β* (i.e. 1 or ∞). Based on previous results reported in the literature for regular stimulation [49, 50], we chose 0.2 Hz as a separation frequency in order to distinguish between two different conditions: time-dependence (MSR > 0.2 Hz) or independence (MSR < 0.2 Hz) of responses to subsequent stimuli. It is possible to note how the network preference for irregular stimulation sequences described above holds true only for fast sequences (i.e. MSR > 0.2 Hz). In the case of slower stimulations frequencies, observed PCs value are almost matching for all filter lengths considered, with regular stimulations providing slightly better (but not significant) matches between inputs and outputs. For faster sequences, a significantly higher I/O correlation can be observed for irregular stimulation sequences, at least for filter lengths including more than a single stimulus on average (i.e. filters windows longer than 2 s). In Fig. 4b we reported the statistical distributions of PCs for all filter lengths: for lower stimulation frequencies, irregular stimulation has largely the same effect of a regular one, while the opposite is true for higher stimulation frequencies. It is possible to notice how the only population resulting in a significant difference from the others is the one of PCs for fastest stimulation sequences in the case for *β = ∞*: as stimulation frequency increases, I/O correlation in the case of regular stimulation seems to degrade, whereas this does not happen for irregular stimulation.

**Fig. 5:**
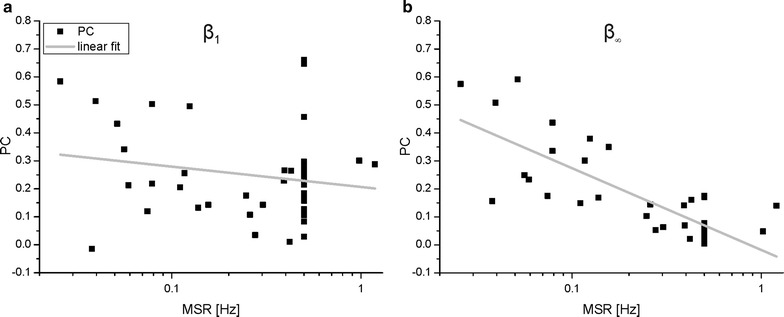
Relation between mean stimulation rate and I/O correlation. In the two scatter plots, each *dot* represents a 10-min stimulation phase. On the *X axis*, the average stimulation rate is presented, while the *Y axis* shows the PC obtained following a pre-filtering with an 8 s long window. At X = 0.5, the relevant PCs from the experiments performed with protocol 1 during STIM1 are represented, while the remaining points are relative to the PCs obtained during the first stimulation sessions of protocol 2. The *gray fitting line* is obtained as a linear interpolation using the natural logarithm of the MSR values as Xs and the PCs as Ys. The fitted slope is −0.031, with 95% confidence bounds at −0.083 and 0.02 (*lower* and *upper*, respectively) and a R^2^ value of 0.0054 for the graph in the *left panel*, relative to β = 1. The fit for the data represented in the graph reported in the *right panel* (β = ∞) results in a slope of −0.13 (95% confidence bounds are −0.16, −0.097) and a R^2^ of 0.66. Data reported in panels (**a**) and (**b**) are included in Additional file 2

Figure 5 provides evidence of neuronal networks progressively losing the capability to provide 1-to-1 responses to stimulation with increasing stimulation rates (thus resulting in a decreasing PC) (cf. Additional file 2), but only in the case of regular stimulation (cf. Fig. 5b). On the other hand, when stimulation intervals follow a 1/*f* distribution (cf. Fig. 5a), stimulation rate has essentially no influence on the observed responses of the network, with correlation values remaining almost constant throughout the analyzed interval (MSR ~ 0.01–1.3 Hz). In order to confirm this, a simple least-squares fitting has been performed to find the best linear fit between the logarithm of MSR in each stimulation phase and the resulting PC (thin gray lines in Fig. 5). This fitting confirms that the linear dependency between MSR and PCs is negligible in the case of *β* = 1: R^2^ is 0.01 and the 95% confidence interval for the slope estimation includes 0. Conversely, for regular stimulation sequences, the MSR has a significant impact on the resulting I/O correlation (R^2^ = 0.66 and the upper bound of the 95% confidence interval for the slope parameter is negative). The graphs of Fig. 5 include the MSR/PC pairs obtained during STIM1 for the experiments conducted with both protocol 1 (i.e. points at X = 0.5) and protocol 2.

## Discussion

The first aim of this work was that of verifying whether dissociated neuronal cultures would present the same preference to irregularity in stimulation as isolated neurons. For this point, a series of experiments was conducted closely matching those performed by Gal and Marom [15] on single neurons. Our results present a good degree of similarity to those described in their work. In fact, neural cultures are mostly entrained by stimulation sequences characterized by the same range of *β* values as single neurons. This can be qualitatively observed on panel a of Fig. 3, with the IFR trace following closely slow variations in the stimulation rate, while regular stimulation results in responses with no resemblance to the input sequence, and white noise stimulation (i.e. *β* = 0) altogether lacks slow fluctuations by design. This observation can be quantitatively appreciated in Fig. 3b, c, with slowly-changing stimulation sequences (i.e. those resulting from *β* values of 0.5, 1 and 1.5) resulting in significantly higher PCs than regular ones. White noise stimulation does not differentiate from scale-free-like stimulation for shorter timescales (e.g. T = 4 s), but provides lower stimulus–response correlations for longer timescales. Furthermore, the most likely (9 out of 18 stimulation sessions) value of *β* resulting in the highest I/O correlation at T = 8 s is 1 (cf. Fig. 3d), as previously observed in Gal’s work for single neurons.

The second set of experiments also resulted in interesting findings: I/O cross correlation decreased with an increase in stimulation rate for regular sequences, as expected, but it remained almost constant in the case of stimulation sequences with *β* value of 1. Previous literature (Gal et al. [32]) reported how single neurons switched from a 1-to-1 response mode to an erratic response pattern as stimulation rates increased above a certain threshold. Our results point out that something similar is true at the network level: well-separated stimuli result in the network equivalent of 1-to-1 responses and thus high I/O cross-correlation values, while stimuli closer together in time result in erratically skipped responses and little resemblance between input and output signals. This behavior breaks down dramatically when the stimulation sequence ceases to be regular and high PCs scores can be observed even at the highest stimulation frequencies considered for *β* values of 1 (cf. Fig. 5).

At the single cell level, different mechanisms might act as causes for the ‘preference’ of neurons for noisy inputs at short time scales. Likely candidates include properties of ion channels or time constants of intracellular ion concentration recovery following spikes [51, 52]. A reasonable, general explanation is that regular stimulation prevents slower processes from completely recovering, causing neuron behavior to be dictated by stochastic, fast processes. Under irregular stimulation, the occasional longer pauses allow for recuperation of slower processes [15]. Figure 5 shows that a similar explanation is plausible also for the behavior observed at the network level: regular stimulation allows the recovery of all involved cellular and also synaptic processes if it is slow enough, while it cannot occur if the stimulation rate increases too much and a mostly stochastic response pattern takes over. On the other hand, irregular stimulation, even at high MSRs, presents occasional long breaks (or at least lower frequency stimulation phases), allowing a more complete recovery to take place, so that the I/O correlation is largely independent from the average rate of stimulation.

In 2013, a paper published by the group of Egert [50] suggested that strength and duration of neural network responses to electrical stimulations followed an exponential saturating profile as a function of the length of the preceding inactivity period. These results were compatible with short-term synaptic depression caused by bursts, due to depletion of readily releasable pool of neurotransmitter vesicles, also in conjunction with GABAergic inhibition (see Discussion of [50]). Also spike-frequency adaptation in single neurons during bursting activity could affect stimulus–response dynamics [53]. These results (and their interpretation) are in agreement with what we found in our experiments: “noisy” (i.e. irregular-frequency) stimulation better entrains cortical networks’ activity because the sequence of time intervals between consecutive stimuli better allows recovery of limited synaptic resources (with respect to fixed, and especially short, inter-stimulus intervals). Similar in vivo studies about state-dependent neuronal responsiveness confirmed what observed by Weihberger et al. [50] (see also [54, 55]), thus allowing to hypothesize that cortical network properties underlying this behavior are retained even in simpler in vitro model systems. Then, we expect that our results could be also reproduced in vivo, and relevant to the design of in vivo stimulation protocols leading to improved predictability and efficiency.

## Conclusions and future perspectives

Our work suggests that regular stimulation sequences are almost ignored by neuronal networks, at least in the sense that the resulting activity bears very little resemblance to the input sequence. This is especially true if the stimulus rate approaches the Hertz range. What this observation suggests, in turn, is that particular care must be taken when designing stimulation patterns for neural systems (be it for research or clinical purposes), as features that are usually overlooked, such as regularity are instead of paramount importance.

A couple of questions are left open. First, we would like to explore the range of stimulation parameters (i.e. amplitude and duration of the stimulus) allowing to either amplify or reduce the correlation between the stimulation and the response train or among spike trains during several repetitions of the stimulation. Secondly, it would be interesting to investigate the exact amount of ‘irregularity’ needed in stimulation sequences in order for them to be effective. In this work we used input sequences with mean stimulation intervals of 2 s and a SD of 0.5 s. How small can the SD be, before the sequence becomes indistinguishable from a regular one? Is the absolute value of noise standard deviation important or rather the ratio between average interval and standard deviation? Finally, adding picrotoxin to our networks would certainly modify the stimulus–response dynamics (as already shown by [50]). Network disinhibition would likely increase the duration of both spontaneous and evoked network bursts (also facilitating their spatio-temporal propagation), but at the same time would deepen synaptic depression, thus increasing the recovery time, as shown by Weihberger et al. [50]. In this context it would be interesting to test our stimulation protocols in the presence of PTX, especially to assess the capability of networks to follow higher stimulation frequencies, even if irregularly distributed. Providing an answer to those questions with future experimental campaigns might result in a better understanding of the features that a signal requires to be correctly represented in a neural system.

## Additional files

**Additional file 1.** Document containing supplementary Figures S1 and S2 and their corresponding captions.

**Additional file 2.** File containing all data used to compute the statistical distributions reported in Figs. 2, 3, 4, and 5 and shown as box plots and bar-graphs.

## Abbreviations

BI: burstiness index
IFR: instantaneous firing rate
ISR: instantaneous stimulation rate
MBR: mean bursting rate
MEAs: micro-electrode arrays
MFR: mean firing rate
MSR: mean stimulation rate
PC: Pearson coefficient
PSD: power spectral density
PSTH: post-stimulus time histogram
