# Supplementary material for: Investigating the impact of electrical stimulation temporal distribution on cortical network responses
Source: BMC Neurosci. 2017 Jun 12;18:49. doi: 10.1186/s12868-017-0366-z (PMC5469148; doi:10.1186/s12868-017-0366-z)
Supplement: Supplementary file 1 — Additional file 1. Document containing supplementary Figures S1 and S2 and their corresponding captions. [file 12868_2017_366_MOESM1_ESM.docx]

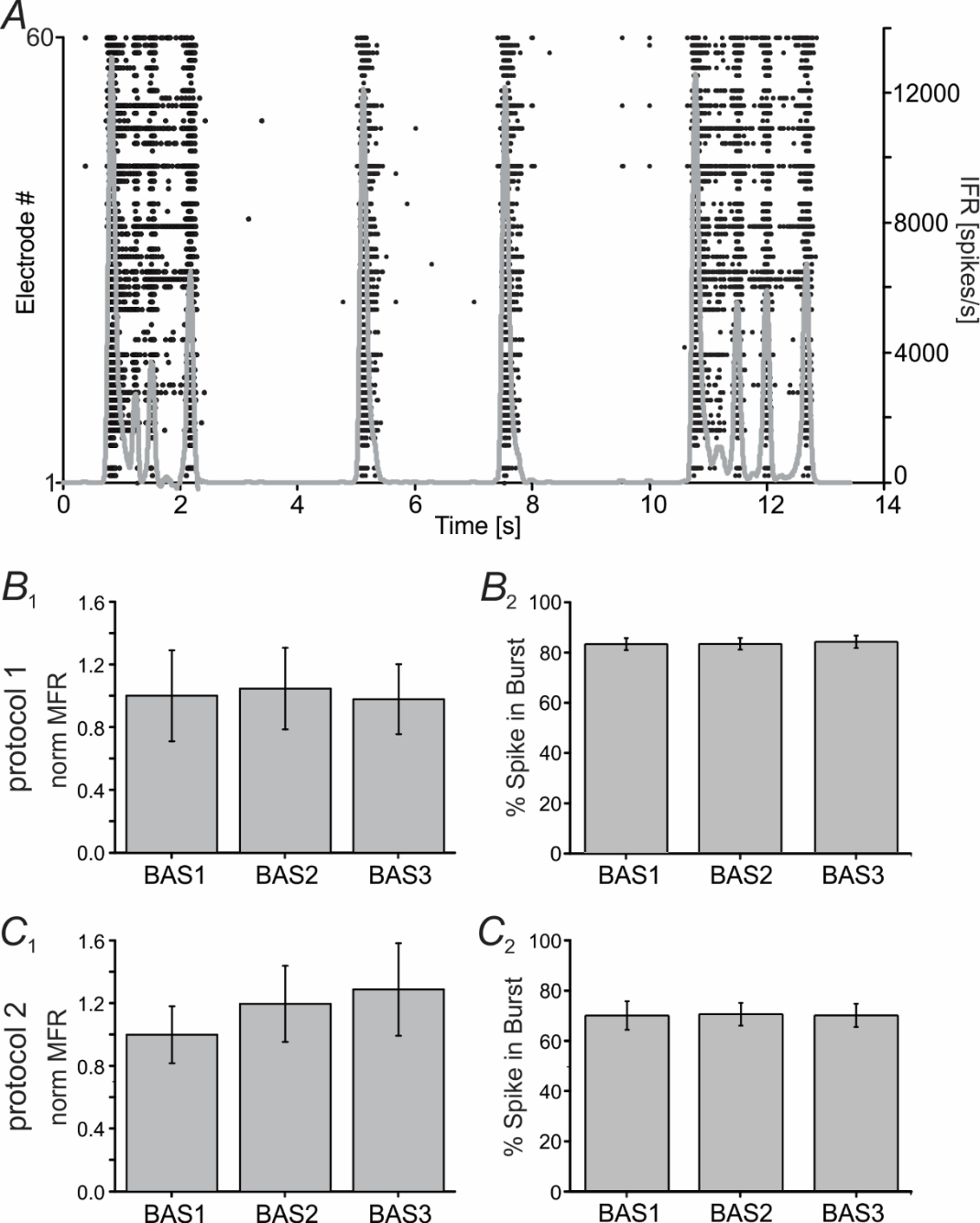


**Figure S1.** **Analysis of spontaneous activity for the two designed protocols.** **A.** Raster plot of 14 seconds of spontaneous activity from 60 electrodes. Each black dot represents a spike. Superimposed trace (in gray) is the not filtered Instantaneous Firing Rate (IFR), obtained with a rectangular kernel of 100s and undersampling factor equal to 10. **B**. Average values of normalized Mean Firing rate (norm MFR) and percentage of spikes within burst for experimental protocol 1. *B1*. Average values of MFR during the three basal phases (BAS1, BAS2, BAS3) of spontaneous activity recording. No statistical difference is observed among the three conditions. *B2*. Average values of the percentage of spikes within burst during the three basal phases (BAS1, BAS2, BAS3) of spontaneous activity recording. No statistical difference is observed among the three conditions. **C**. MFR and percentage of spikes within burst for experimental protocol 2. *C1*. Average values of normalized MFR during the three basal phases (BAS1, BAS2, BAS3) of spontaneous activity recording. No statistical difference is observed among the three. *C2*. Average values of BI during the three basal phases (BAS1, BAS2, BAS3) of spontaneous activity recording. No statistical difference is observed among the three conditions. Data are presented as mean ± se. Statistical analysis: Kruskal-Wallis Analysis of Variance on Ranks, *p<0.05, post-hoc comparison with Tukey’s method.


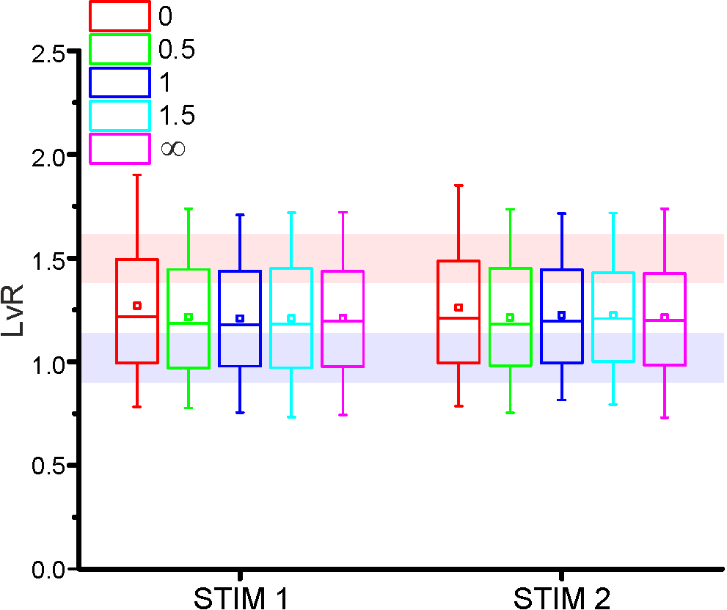


**Figure S2. Statistical distributions of local variation with refractoriness (LvR) parameter of firing activity during stimulation phases (i.e. STIM1 and STIM2), as a function of β** (see color legend). We did not find any statistically significant difference among different β values, neither in STIM1 or STIM2 (Kruskal-Wallis ANOVA on ranks, p-level = 0.05). The light blue square highlights the LvR range of RANDOM activity, whereas the pink square depicts the LvR range of BURSTY activity. The activity of our cortical networks lies in between these two firing regimes.
